# Biological Augmentation With Retro-Drilling Core Decompression in Early Stage of Femoral Head Avascular Necrosis

**DOI:** 10.1016/j.eats.2024.103093

**Published:** 2024-06-21

**Authors:** Murat Bozkurt, Enejd Veizi, Neslihan Fırat, Ali Şahin

**Affiliations:** a Department of Orthopedics and Traumatology, Ankara Acıbadem Hospital, Ankara, Turkey; b Department of Orthopedics and Traumatology, Ankara Bilkent City Hospital, Ankara Yıldırım Beyazıt University, Ankara, Turkey; c Institute of Health Sciences, Physiotherapy and Rehabilitation, Lokman Hekim University, Ankara, Turkey; d Department of Orthopedics and Traumatology, Ankara Etlik City Hospital, Ankara, Turkey

## Abstract

Osteonecrosis of the femoral head can lead to end-stage osteoarthritis when left untreated. The incidence has been on the rise since the onset of the COVID-19 pandemic. Core decompression of the femoral head is usually the first line of surgical treatment when conservative options fail. Additional biologic support (e.g., bone marrow aspiration concentrates, mesenchymal stem cell derivatives, adipose-derived stromal vascular fraction) has been shown to augment the effects of core decompression alone, but the nature and amount of this additional support is still a topic for debate. This technique describes a surgical approach featuring debridement through retro-drilling, core decompression, and biologic augmentation with stromal vascular fraction and bone marrow aspiration concentrate on the early stages of osteonecrosis of the femoral head.

Osteonecrosis of the femoral head (ONFH) is a progressive and debilitating disease that often leads to end-stage osteoarthritis.^1^^,^^2^ Disruption of local blood flow to the femoral head, subsequent increase in intraosseous pressure, and final necrosis are thought to be behind the cause of the disease. The incidence varies between 5,000 and 20,000 cases per year worldwide, with a marked increase since the onset of the COVID-19 pandemic and the widespread use of corticosteroids.^2^^,^^3^ Conservative treatment, whether physiotherapy or medication alone, has been shown to have little effect on disease progression, so surgical treatment is generally the treatment of choice, especially in symptomatic cases.^4^^,^^5^

Core decompression of the femoral head is usually the first choice of surgical treatment for ONFH cases.^1^^,^^6^ Since its original conception by Hernigou et al.,^7^ the procedure itself has undergone a number of modifications, including the addition of intraosseous injections (bone marrow aspiration concentrates [BMACs], mesenchymal stem cell derivatives, and adipose-derived stem cells), vascularized fibula autografts, tantalum rods, and other various artificial materials.^1^^,^^2^^,^^4^^,^^6^^,^^8^ The rationale for additional intraosseous support is based on the notion that once the necrotic area is debrided and drained, it will no longer have the necessary structural support and therefore may be prone to preemptive collapse, leading to early conversion to total hip arthroplasty.^9^^,^^10^

Adipose-derived stromal vascular fraction (SVF) is obtained from lipoaspirate and requires no cell culture.^11^^,^^12^ With its heterogeneous cell population consisting of adipose stromal cells, hematopoietic stem cells, progenitor cells, preadipocytes, fibroblasts, pericytes, macrophages, lymphocytes, and endothelial cells, among others, SVF has gained attention these past years as a possible alternative to adipose and mesenchymal stem cells for the treatment of ONFH due to obvious lesser ethical concerns.^12^ The aim of this article, therefore, is to describe a different surgical technique for the debridement through retro-drilling, core decompression, and biologic augmentation with SVF of the early stages of ONFH.

## Surgical Technique

The technique is described in Video 1. The patient is placed supine on a radiolucent table. After anesthesia, a pre-emptive fluoroscopic check is performed on the hip joint, and anteroposterior and lateral views are obtained. After proper skin preparation, the indexed lower extremity is draped and covered in a sterile way, with an exposed ipsilateral iliac crest.

### BMAC

**Fig 1:**
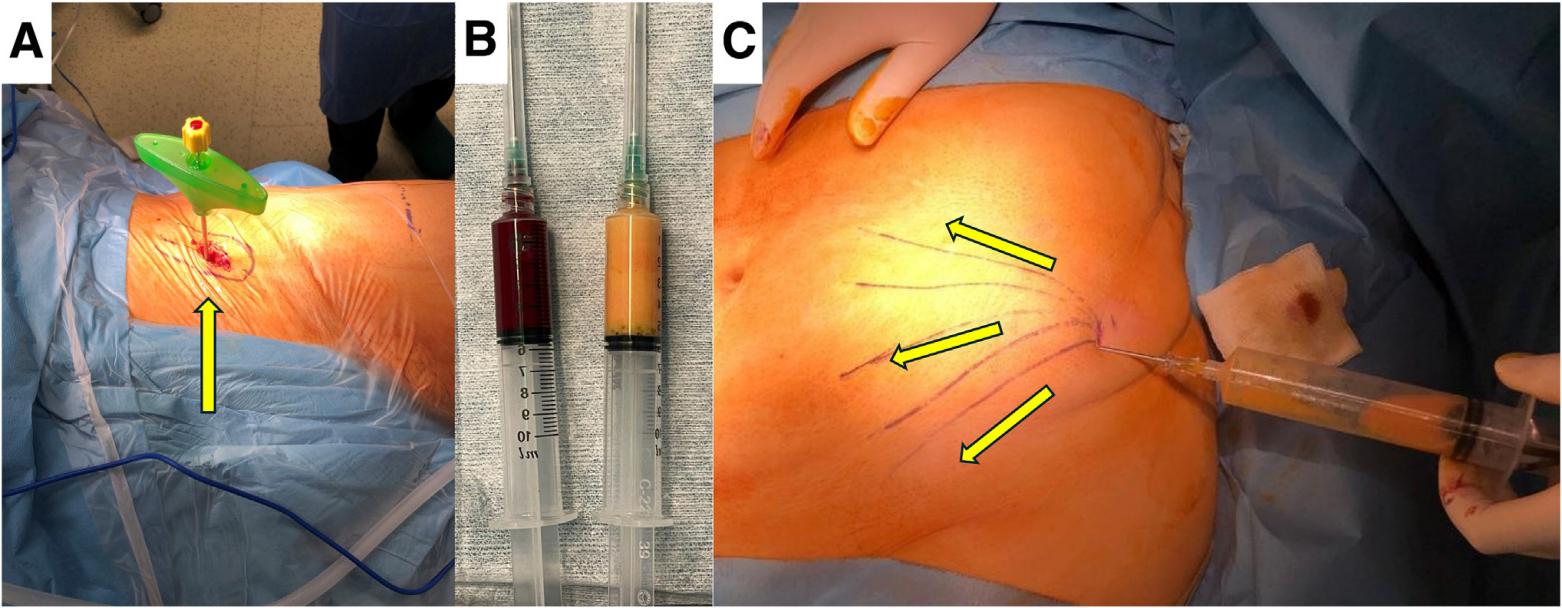
The patient is placed supine and the ipsilateral hip together with a portion of the lower abdomen is prepared and draped in a sterile fashion. (A) The procedure in this case is a left hip with the patient’s head facing on the left side. (B) Using a thick needle (arrow), approximately 60 mL of bone marrow is aspirated and concentrated into bone marrow aspiration concentrate (left). (C) For the lipoaspirate, a small stabbing skin incision is performed on the abdomen and 60 mL of adipose tissue is obtained though lipoaspiration (arrows) (left hip, patient laying supine with their head being in the upper part of the figure). The aspirate is then processed into stromal vascular fraction (B, right).

A bone marrow aspiration needle is inserted into the iliac crest and approximately 60 mL of bone marrow is aspirated. The obtained marrow is concentrated into BMAC using the Angel BMAC Cellular Therapy System (Bone Marrow Concentrate, Angel System; Arthrex) (Fig 1).

### SVF

A small stabbing skin incision is performed in the abdominal area, lateral and inferior to the umbilicus, and 60 mL of adipose tissue is obtained through liposuction. The tissue is then processed using the Autologous Conditioned Adipose SVF system (Arthrex) (Fig 1).

### Bone Graft Harvesting

**Fig 2:**
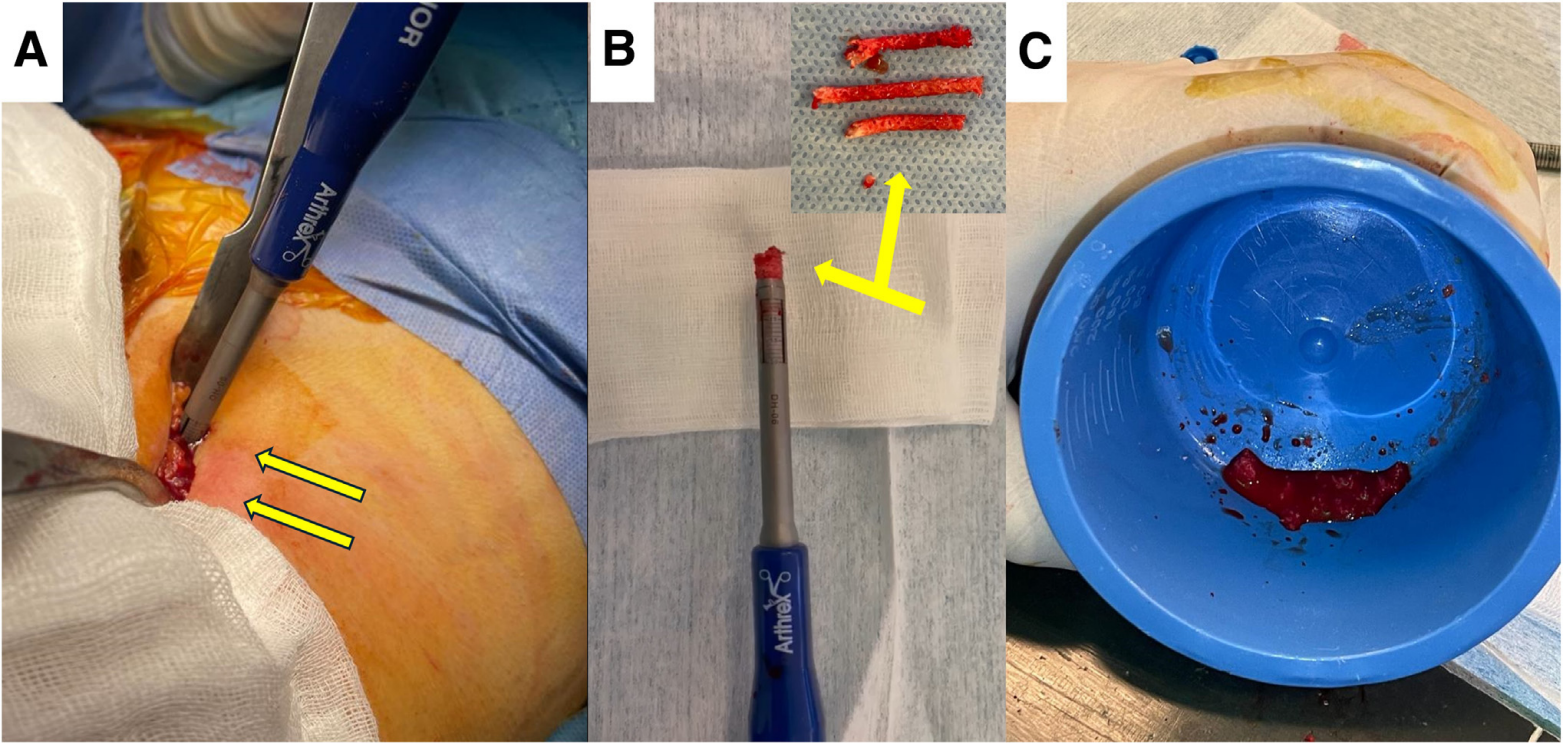
(A) Through an incision on the iliac crest (left hip, patient supine), small cylindrical cortical autograft fragments are obtained using a thick bone marrow aspiration needle or an osteochondral graft harvester (arrows). The grafts are carefully extracted (arrows) (B) while 80% of this obtained graft is crushed until a putty consistency is achieved and mixed with a small portion of the obtained bone marrow aspiration concentrate and stromal vascular fraction (C). The rest is kept intact and will be used to seal the decompression tunnel.

An additional small skin incision is performed on the ipsilateral iliac crest. After proper exposure of the osseous tissue, small cortical autograft fragments are obtained using a thick bone marrow aspiration needle or an osteochondral graft harvester. The obtained cylindrical cortical autograft fragments are carefully extracted, with 80% of them crushed until a putty consistency is achieved. The rest is kept intact and will be used to seal the decompression tunnel (Fig 2).

### Preparation of Injectable Mixture

The BMAC and SVF aspirates are mixed together through the double injector technique from Arthrex. Five to 10 drops of the final mixture are added to reinforce the obtained cortical autograft in the side cup.

### Core Decompression Procedure

**Fig 3:**
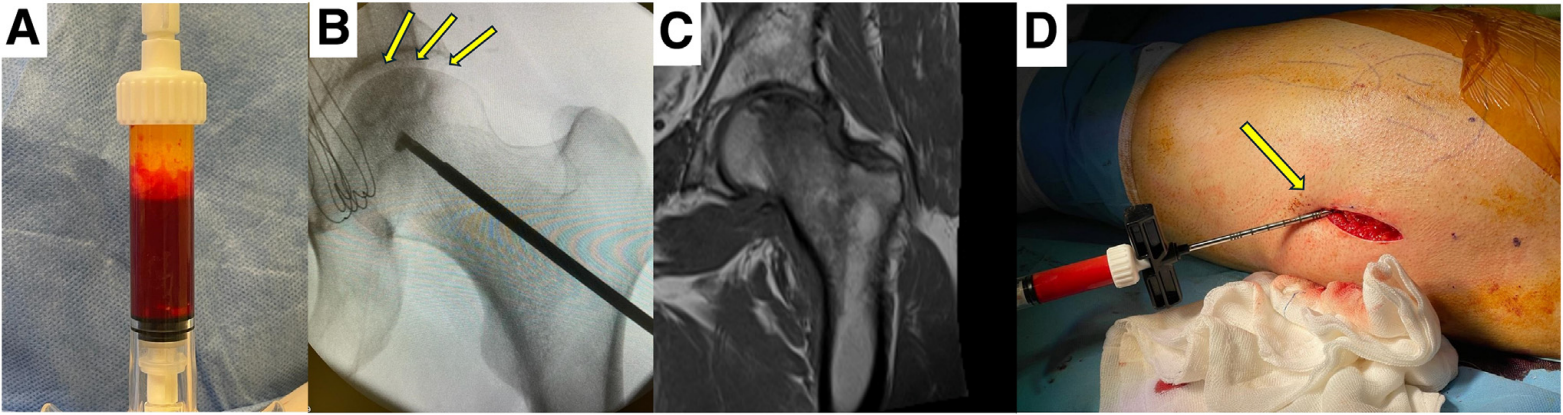
(A) The bone marrow aspiration concentrate (BMAC) and stromal vascular fraction (SVF) aspirates are mixed. (B, C) The decompression procedure is achieved through a guidewire and retro-drilling to decompress and debride the osteonecrotic and edematous region, according to the intraoperative fluoroscopic and magnetic resonance imaging views (arrows). (D) A thin cannula is then placed at the entrance of the decompression tunnel (arrow), and the previously obtained crushed autografts together with the BMAC and SVF mixture are gently injected into the debrided area. The drill entrance is then sealed with the intact fragment of the cortical graft (left hip, patient supine, caudad to cephalad from left to right).

The lateral cortex of the index hip joint is accessed through a skin incision, and Hohmann retractors are used to obtain good bony exposure. A guidewire is advanced through fluoroscopic guidance to the planned and confirmed osteonecrotic area of the femoral head. The drill guide of the IntraOsseous BioPlasty Technique Set (Arthrex) is advanced and the final position is confirmed. A flip-cutter retro-drill is then used to decompress and debride the osteonecrotic region. An additional radio-opaque solution injection can be used to check the debrided area. Upon achieving the desired debridement amount, the drill is removed, and the surgical table is partially tilted sideways so that the index hip faces upward. A thin cannula is then placed at the entrance of the decompression tunnel and the previously obtained cortical autografts are gently hammered into the debrided area. The BMAC and SVF mixture is subsequently injected, and the drill entrance is closed with a final fragment of cortical graft. The skin flaps are then closed in a standard fashion and the procedure is concluded (Fig 3).

### Postoperative Rehabilitation Protocol

All patients were prescribed ibandronic acid 150 mg (once a month), aspirin 100 mg (once a day), hyperbaric oxygen therapy (30 sessions), atorvastatin 20 mg (once a day), and oral vitamin D 20,000 IU (twice a week), for a total of 3 months after surgery. They are mobilized the following day with a nonweightbearing protocol for the first 6 weeks. Range of motion and gluteus strengthening exercises are started on the second postoperative week.

### Indications and Contraindications

This procedure is indicated for all precollapse ONFH cases (Ficat-Arlet stages 1 and 2A). Collapse of the necrotic area is a strict contraindication and may accelerate the overall joint degeneration (Table 1 and Table 2).

**Table 1:** Pearls and Pitfalls of the Technique

Pearls
• The surgeon should use fluoroscopy throughout the case for guidance. • A change in the mallet ping pitch is noted when the trocar reaches the necrotic bone. • The BMAC and SVF should be inoculated in the area of necrosis through the syringe placed into the cannula. • Postoperatively, weight bearing as tolerated, with crutches, is immediately initiated.
Pitfalls
• Failure to use frequent fluoroscopic imaging can result in joint violation from advancing the trocar too far. • Failure to add heparin to the syringes and prime the needles with anticoagulant can result in clot formation when extracting the bone marrow. • If resistance is present, the surgeon should withdraw the cannula by a couple of millimeters and reattempt inoculation of the biologic mixture.

BMAC, bone marrow aspiration concentrate; SVF, stromal vascular fraction.

**Table 2:** Advantages and Disadvantages of the Procedure

Advantages
• The procedure is minimally invasive. • The patient’s own bone marrow and adipose tissue are used to stimulate biological healing. • The BMAC and SVF mixture is inoculated directly in the area of necrosis.
Limitations
• The procedure is not indicated in later stages of avascular necrosis. It is not safe to perform without the aid of intraoperative imaging. • General anesthesia is frequently required.

BMAC, bone marrow aspiration concentrate; SVF, stromal vascular fraction.

## Discussion

Treatment of ONFH is challenging because of the unpredictability of necrotic subchondral bone.^3^^,^^13^ Since core decompression has been defined, the possibility of creating an intraosseous cavity and risking preemptive collapse has been pointed out.^2^^,^^6^^,^^7^ Several artificial (e.g., tantalum rods) and biological (e.g., vascularized bone autograft) augmentation techniques have been described in the literature, with little or no difference between them, with the matter still being a topic of debate.^4^^,^^14^^,^^15^ The technique described here offers a different approach to an old dilemma with a relatively less invasive approach. We hypothesize that the addition of small fragments of cortical bone to the debrided and decompressed necrotic area will help provide additional mechanical support to a vulnerable region.

Despite the extensive literature published in its favor, core decompression, alone or in combination, does not solve the problems associated with angiogenesis and bone reconstruction in the necrotic area.^9^^,^^15^ Bone marrow aspirate concentrate was introduced as a possible solution due to the ability of stem cells to differentiate into various cell lineages, including osteoprogenitor cells.^16^ Hernigou et al.^17^ introduced the concept in 2006, and since then, several studies and meta-analyses have confirmed the superior results of combined therapy of core decompression and BMAC injection in the medium- and long-term follow-up. Wang et al.^6^ found that the combined use of core decompression and autologous BMAC resulted in better pain relief and clinical outcomes compared with core decompression alone and could effectively delay femoral head collapse.

The SVF, on the other hand, has gained increasing attention in the past decade because it can be readily obtained from lipoaspirates after enzymatic digestion without cell culture.^2^^,^^12^ Numerous studies have confirmed its similarity and often superiority to BMAC in the treatment of knee osteoarthritis, tendon healing, and osteonecrosis. In a recent meta-analysis on knee osteoarthritis, Bolia et al.^13^ found that a single injection of BMAC or SVF into the knee joint resulted in symptomatic improvement at short-term follow-up, but SVF was more effective than BMAC. Aydın et al.^11^ investigated the effects of a mixture of BMAC and SVF on the healing of Achilles tendon rupture and found that the combined application of BMAC and SVF improved healing compared with the individual application of each mixture. Based on these results, our approach to ONFH aims to increase the likelihood of long-term femoral head survival while improving mechanical support.

Despite its possible ambitious effects, the technique has its own limitations. Clinical experience and several studies have shown that core decompression, with or without augmentation, gives better results only in the precollapse stages of ONFH, making this technique unsuitable for advanced osteonecrosis cases. Another disadvantage could be related to the cost of the procedure, which makes it difficult to access despite the correct diagnosis. Finally, the technique requires an additional stab incision at the iliac crest and the harvesting of cortical autograft. Although the amount of graft removed is small, access to the iliac crest has been shown to result in residual pain in the aftermath of surgery.

## Disclosures

All authors (M.B., E.V., N.F., A.S.) declare that they have no known competing financial interests or personal relationships that could have appeared to influence the work reported in this paper.

## Supplementary Data

Video 1 Left hip: The patient is placed supine on the surgical table, and after proper skin preparation, the indexed thigh is draped with an exposed ipsilateral iliac crest and lower abdomen. Using a thick needle, approximately 60 mL of bone marrow is aspirated and concentrated into bone marrow aspiration concentrate (BMAC). For the lipoaspirate, a small stabbing skin incision is performed on the abdomen and 60 mL of adipose tissue is obtained though lipoaspiration. The aspirate is then processed into the stromal vascular fraction (SVF). Through an incision on the ipsilateral iliac crest, small cylindrical cortical autograft fragments are obtained using an osteochondral graft harvester. The grafts are carefully extracted while 80% of this obtained graft is crushed until a putty consistency is achieved and mixed with a small portion of the obtained BMAC and SVF. The rest is kept intact and will be used to seal the decompression tunnel. The BMAC and SVF aspirates are mixed. The decompression procedure follows through a guidewire and retro-drilling to decompress and debride the osteonecrotic region. A thin cannula is then placed at the entrance of the decompression tunnel, and the previously obtained crushed autografts together with the BMAC and SVF mixture are gently injected into the debrided area. The drill entrance is then sealed with the intact fragment of the cortical graft.

## References

[1] Salas A.P., Mazek J., O’Donnell J. (2021). Hip arthroscopy and core decompression for avascular necrosis of the femoral head using a specific aiming guide: A step-by-step surgical technique. Arthrosc Tech.

[2] Zhu S., Zhang X., Chen X. (2021). Comparison of cell therapy and other novel adjunctive therapies combined with core decompression for the treatment of osteonecrosis of the femoral head: A systematic review and meta-analysis of 20 studies. Bone Joint Res.

[3] Veizi E., Erdoğan Y., Sezgin B.S. (2023). The painful joint after COVID-19 treatment: A study on joint osteonecrosis following COVID-19-related corticosteroid use. Jt Dis Relat Surg.

[4] Einhorn T.A., Anoushiravani A.A., Chen K.K. (2019). Osteonecrosis of the femoral head: Can arthroplasty be avoided—a brief review of common interventions. J Hip Surg.

[5] Yurek J.W., Caldwell P.E., Peterson E.E., Pearson S.E. (2023). Arthroscopic-assisted intraosseous bioplasty of the humeral head for osteonecrosis. Arthrosc Tech.

[6] Wang Z., Sun Q.M., Zhang F.Q. (2019). Core decompression combined with autologous bone marrow stem cells versus core decompression alone for patients with osteonecrosis of the femoral head: A meta-analysis. Int J Surg.

[7] Hernigou P., Mathieu G., Poignard A. (2006). Percutaneous autologous bone-marrow grafting for nonunions. Surgical technique. J Bone Joint Surg Am.

[8] Gupta A.K., Frank R.M., Harris J.D. (2014). Arthroscopic-assisted core decompression for osteonecrosis of the femoral head. Arthrosc Tech.

[9] Andronic O., Hincapié C.A., Burkhard M.D. (2021). Lack of conclusive evidence of the benefit of biologic augmentation in core decompression for nontraumatic osteonecrosis of the femoral head: A systematic review. Arthroscopy.

[10] Arbeloa-Gutierrez L., Dean C.S., Chahla J., Pascual-Garrido C. (2016). Core decompression augmented with autologous bone marrow aspiration concentrate for early avascular necrosis of the femoral head. Arthrosc Tech.

[11] Aydın E.Y., Aşık M., Aydın H.M. (2023). The co-use of stromal vascular fraction and bone marrow concentrate for tendon healing. Curr Stem Cell Res Ther.

[12] Hong Z., Zhang Y., Chen J., Bi Q. (2023). Adipose-derived stromal vascular fraction injection following core decompression and biochemistry artificial bone graft implantation in osteonecrosis of the femoral head. Int Orthop.

[13] Bolia I.K., Bougioukli S., Hill W.J. (2022). Clinical efficacy of bone marrow aspirate concentrate versus stromal vascular fraction injection in patients with knee osteoarthritis: A systematic review and meta-analysis. Am J Sports Med.

[14] Alkan H., Veizi E., Erdoğan Y. (2023). A combination therapy for osteonecrosis of the femoral head and short-term results. Arch Basic Clin Res.

[15] Liu Q., Guo W., Li R., Lee J.H. (2021). Efficacy of various core decompression techniques versus non-operative treatment for osteonecrosis of the femoral head: A systemic review and network meta-analysis of randomized controlled trials. BMC Musculoskelet Disord.

[16] Oryan A., Kamali A., Moshiri A., Baghaban Eslaminejad M. (2017). Role of mesenchymal stem cells in bone regenerative medicine: What is the evidence?. Cells Tissues Organs.

[17] Hernigou P., Habibi A., Bachir D., Galacteros F. (2006). The natural history of asymptomatic osteonecrosis of the femoral head in adults with sickle cell disease. J Bone Joint Surg Am.

